# Inhibition of Non-Small Cell Lung Cancer Proliferation and Survival by Rosemary Extract Is Associated with Activation of ERK and AMPK

**DOI:** 10.3390/life12010052

**Published:** 2021-12-31

**Authors:** Eric J. O’Neill, Jessy Moore, Joon Song, Evangelia Litsa Tsiani

**Affiliations:** 1 Department of Health Sciences, Brock University, St. Catharines, ON L2S 3A1, Canada; eo15nv@brocku.ca (E.J.O.); jmoore6@brocku.ca (J.M.); joon1@ualberta.ca (J.S.); 2 Centre for Bone and Muscle Health, Brock University, St. Catharines, ON L2S 3A1, Canada

**Keywords:** lung cancer, rosemary extract, ERK, AMPK, apoptosis, polyphenolics, mTOR, p70S6K

## Abstract

Non-small cell lung cancer (NSCLC) represents an aggressive form of lung cancer which often develops resistance to chemo- and radiotherapy emphasizing a need to identify novel treatment agents to combat it. Many plants contain compounds with anti-inflammatory, antimicrobial, antidiabetic, and anticancer properties and some plant-derived chemicals are used in the treatment of cancer. A limited number of in vitro and in vivo animal studies provide evidence of anticancer effects of rosemary (*Rosmarinus officinalis*) extract (RE); however, no studies have explored its role in H1299 NSCLC cells, and its underlying mechanism(s) of action are not understood. The current study examined the effects of RE on H1299 cell proliferation, survival, and migration using specific assays. Additionally, immunoblotting was used to investigate the effects of RE treatment on signalling molecules implicated in cell growth and survival. Treatment with RE dose-dependently inhibited H1299 proliferation with an IC_50_ value of 19 µg/mL. Similarly, RE dose-dependently reduced cell survival, and this reduction correlated with increased levels of cleaved poly (ADP-ribose) polymerase (PARP), a marker of apoptosis. RE was also able to inhibit cell migration as assessed with a wound healing assay. These cellular effects of RE were associated with an increase in phosphorylated levels of extracellular signal-regulated kinase (ERK), AMP-activated protein kinase (AMPK), and its downstream targets ACC, the mTORC1 protein raptor, and decreased p70S6K phosphorylation. More studies are required to fully examine the effects of RE against NSCLC.

## 1. Introduction

Lung cancer was the leading cause of cancer-related death in 2020, and non-small cell lung cancer (NSCLC) accounts for 80–85% of all lung cancer cases in North America [1,2]. NSCLC is very aggressive with fewer than 20% of diagnosed individuals surviving five years despite the use of aggressive chemo- and radiation therapies [2]. Resistance of NSCLC to current therapies is a growing concern; therefore, exploration into new treatment options for this aggressive form of cancer is necessary [2].

Historically, many plants have been the source for isolation of compounds that are used in the treatment of cancer. For example, extracts of European (*Taxus baccata*) and pacific (*Taxus brevifolia*) yew are the sources of docetaxel and paclitaxel, which are common chemotherapeutic drugs used in the treatment of breast, lung, and ovarian cancers [3,4]. Rosemary extract (RE) has been shown to exhibit anticancer properties including inhibition of cancer cell proliferation and survival, and enhanced apoptotic activity in vitro and in vivo in colon, breast, prostate, and leukemic cancers, among others [5,6]. However, limited data exist regarding the effects of RE in lung cancer and little is known about the signalling mechanisms underlying its antiproliferative properties. Rosemary contains up to 57 polyphenolic compounds including rosmarinic acid (RA), carnosic acid (CA), and carnosol (COH) [7]. Foods/plants rich in polyphenols have promising chemotherapeutic potential and further investigation of polyphenol-rich plant extracts, such as rosemary, may lead to the discovery of molecules with potent anticancer properties and improve knowledge of their anticancer mechanisms.

Cancer is characterized by sustained proliferative signalling, evasion of growth suppressors, activation of invasion and metastasis, replicative immortality, induction of angiogenesis, and resistance to cell death [8]. There are several intracellular signalling pathways that may become dysregulated in cancer cells and give rise to these characteristics. One commonly dysregulated pathway in cancer is the Ras-Raf-MEK-ERK cascade, which is often implicated in the enhanced proliferation and survival of cancer cells [9]. Activated extracellular signal-regulated kinase (ERK) modifies various substrates that govern cell growth, differentiation, and survival; and modulates cytosolic targets and nuclear transcription factors such as CREB, ELK-1, ETS, NF-κB, and c-Myc [10].

The energy sensing 5′-adenosine monophosphate (AMP)-activated protein kinase (AMPK) is important in regulating cellular energy balance and protecting cells under conditions of metabolic stress. AMPK is a highly conserved heterotrimeric kinase complex composed of a catalytic α subunit and two regulatory (β and γ) subunits [11,12,13]. Activation of AMPK occurs when there is an increased concentration of AMP and a fall in ATP within the cell. This is achieved through phosphorylation of AMPK by the tumour suppressor liver kinase B1 (LKB1), or by the calcium/calmodulin-dependent protein kinase kinase β (CaMKKβ). LKB1 is a tumor suppressor and its deficiency can promote the formation of tumours as is the case in Peutz–Jeghers syndrome [13]. Once activated, AMPK inhibits anabolic processes and protein synthesis by inhibiting mTOR [13]. AMPK also phosphorylates and inhibits acetyl CoA carboxylase (ACC), a key regulator of fatty acid and glycerol lipid synthesis.

Evidence indicate that AMPK may be an important target for cancer prevention and treatment [13,14,15,16,17,18]. Activation of AMPK using AICAR or A-769662 demonstrated anticancer properties in pancreatic cancer cells [14]. AICAR-induced activation of AMPK also improved sensitivity of prostate cancer (PC3) cells to ionizing radiation [15]. Activating AMPK using metformin, a mitochondrial complex I inhibitor, may be able to overcome breast cancer resistance to HER2 inhibitors while decreasing the risk of cardiotoxicity [16]. Additionally, metformin-induced activation of AMPK in lung cancer cells has been shown to inhibit growth and enhance radio-sensitivity [17]. The anticancer effects of many natural products have also been associated with AMPK activation [18]. Resveratrol from red grapes, berberine from plants in the genus *Berberis*, quercetin from many fruits and vegetables, curcumin from turmeric, and epigallocatechin gallate from green tea have all demonstrated anticancer properties that are associated with the activation of AMPK [18]. Downstream of AMPK, ACC is also an important target in cancer therapy because of its involvement in regulation of lipid metabolism. There are significant differences in lipid dependence between normal and cancerous cells and overexpression of ACC in many tumours is indicative of tumour progression and a malignant phenotype [19]. Activation of AMPK can phosphorylate and inhibit ACC activity, thereby reducing lipogenesis in cancer cells leading to decreased cell survival and increased apoptosis [19].

Mammalian target of rapamycin (mTOR) is a member of the phosphatidylinositol 3-kinase (PI3K)-related kinase family and it exists in two structurally and functionally distinct protein complexes: mTOR complex 1 (mTORC1) and mTOR complex 2 (mTORC2). Common to both complexes are mTOR which is responsible for the kinase activity of the complex, and G protein beta subunit-like (GβL), which stimulates the kinase activity of mTOR [20]. mTORC1 integrates growth factor and nutrient signalling to regulate growth and metabolism based on energy levels and nutrient availability. PI3K/Akt signalling activates mTORC1 kinase activity by inhibiting tuberous sclerosis complex 2 (TSC2) allowing rheb to phosphorylate/activate mTOR at ser2448 [21,22]. Activation of mTORC1 promotes growth through inhibition of autophagy via inhibition of ULK1, activation of protein synthesis via p70 S6 kinase (p70 S6K) and eIF4E, and activation of nucleotide synthesis via p70S6K [23]. The regulatory protein associated with mTOR (raptor) is unique to mTORC1. Raptor allows mTORC1 to bind and interact with its downstream substrates [24]. AMPK activation inhibits mTORC1 through phosphorylation/inhibition of raptor at ser722/792 residues. Activated AMPK also causes phosphorylation/activation of TSC2 leading to inhibition of rheb and inhibition of mTORC1 [25]. The function of mTORC2 is less understood than mTORC1. mTORC2 is activated by growth factor signalling and promotes cell proliferation and survival via the Akt pathway. Instead of raptor, mTORC2 has the rapamycin-insensitive companion of mTOR (rictor) which is necessary for ser473 phosphorylation of Akt [26]. The mTOR pathway is often dysregulated in cancer and recently, activating mutations of mTOR were identified in several human cancers making mTOR a potential therapeutic target for cancer treatment [27,28].

Despite evidence supporting the anticancer potential of RE, limited data exist regarding its effects in lung cancer, and little is known about the signalling mechanisms responsible for its anticancer properties. In a previous study, we found inhibition of proliferation and survival of A549 NSCLC cells by RE [29]. In the present study, we investigate the effects of RE in H1299 cells. These cells, unlike A549 NSCLC cells, express the AMPK-activating protein LKB1 and lack the tumour suppressor p53. Similar to A549 cells, the H2199 cell line also has a mutated Ras oncogene (NRAS). Exploring the anticancer potential of RE in various NSCLC cell lines represents a valuable opportunity to understand the different mechanisms involved in RE’s antiproliferative activity and its effectiveness as a potential therapy in cancers with varying tumour mutations.

## 2. Materials and Methods

### 2.1. Materials

Human H1299 NSCLC cells were obtained from American Type Culture Collection (ATCC). Cell culture (RPMI) media, fetal bovine serum (FBS), trypsin, and antibiotic were from GIBCO (Burlington, ON, Canada). Antibodies against total or phosphorylated (ERK, AMPK, ACC, mTOR, Raptor, and p70 S6K), PARP, β-actin, and γ-tubulin were purchased from Cell Signaling Technology via New England Biolabs (Mississauga, ON, Canada). Bovine serum albumin, dimethyl sulfoxide (DMSO), methylene blue and crystal violet stain, mitomycin C (MMC), paclitaxel, and resveratrol were purchased from Millipore Sigma (Oakville, ON, Canada).

### 2.2. Rosemary Extract Preparation

Rosemary extract was prepared as described previously [30] utilizing dried rosemary leaves purchased from Sobeys (Mississauga, ON, Canada) and a methanol extraction method. The final extract was collected by rotary evaporation and stored at −20 °C, protected from light, until use. The extract was solubilized in DMSO (stock concentration 100 mg/mL). This stock solution was used in preparing a working RE solution utilizing cell culture media and the cells were never exposed to more than 0.1% DMSO in any treatment.

### 2.3. Cell Culture and Treatment

H1299 cells were grown in RPMI media supplemented with 10% (*v*/*v*) FBS, and 1% (*v*/*v*) antibiotic-antimycotic solution in a humidified atmosphere of 5% CO_2_ at 37 °C. The final concentration and the time of exposure to RE, resveratrol, or DMSO are indicated in each figure.

### 2.4. Cell Proliferation Assay

The crystal violet cell proliferation assay was performed as described previously [31]. Cells were seeded (1000 cells/well) in triplicate in 96-well plates and treated with the indicated concentration of RE, resveratrol, or DMSO (vehicle control) for 72 h. Fixing and staining with crystal violet dye was performed, and after drying, the dye was solubilized. Absorbance was read at 570 nm using a KC4 plate reader (Bio-Tek). The data are expressed as percent of control.

### 2.5. Clonogenic Survival Assay

Clonogenic assays were performed as described previously [29]. Cells were seeded in triplicate in 6-well plates (800 cells /well), allowed to adhere overnight and exposed to media containing the indicated concentrations of RE for 7 days. Fixing and staining with 0.05% methylene blue was performed and colonies (>50 cells) were counted. The data are expressed as the surviving fraction compared to control.

### 2.6. Immunoblotting

Immunoblotting was performed as described previously [29]. All cells to be used in an individual experiment were seeded on the same day and grown to 90% confluence. The 48 h group was treated first, the 24 h group was treated 24 h later, and then all cells were lysed 24 h later so that all cells were in culture for the same time. Following treatment, the cells were washed with ice cold PBS solution and then lysed with ice cold lysis buffer. Proteins (20 µg) were separated using SDS-PAGE, transferred to a PVDF membrane and incubated with the indicated antibodies. Membranes were stripped and re-probed for β-actin or γ-tubulin. A LI-COR C-Digit blot scanner (LI-COR Biosciences) and corresponding software were utilized to visualize the corresponding bands. Densitometric analysis was performed using Image J software. The data, expressed in arbitrary densitometric units, are presented as the mean ± SEM of control.

### 2.7. Wound Healing Assay

The wound healing assay was performed as described previously [32]. Briefly, H1299 cells were seeded into a 24-well plate and grown to 90–100% confluency. Once confluent, cells were incubated with MMC (1 µg/mL) for 1 h to inhibit proliferation and then a vertical line was drawn down the centre of the well with a pipette tip. The media was removed and the cells were washed twice with 1× PBS followed by treatment with RE. Marks were drawn on the bottom of the plate as a reference for photos. Photos were taken at 0 and 20 h timepoints. The area of the wound was measured with ImageJ software and wound closure percentage was determined as: $\frac{\left(0\;h\;Area\right)-\left(20\;h\;Area\right)}{0\;h\;Area}\times 100\%$.

### 2.8. Statistical Analysis

The data are expressed as the mean ± SEM of the indicated number of independent experiments. Graphpad Prism 6 software was used for statistical analysis and significance was assumed at *p* < 0.05. Analysis of variance (ANOVA) with Dunnett’s post hoc test was used.

## 3. Results

### 3.1. RE Inhibits Proliferation and Survival and Promotes Apoptosis of H1299 Lung Cancer Cells

**Figure 1:**
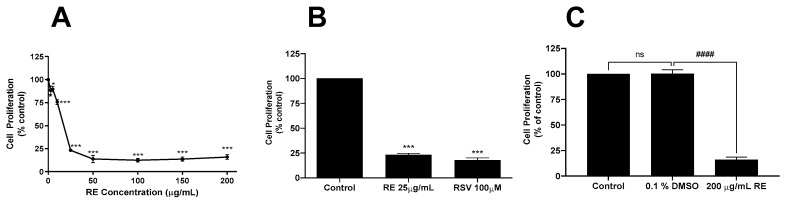
Effect of RE on H1299 cell proliferation. H1299 cells were seeded in triplicate and treated without (control, 0) or with the indicated concentrations of RE (**A**–**C**), resveratrol (RSV; **B**) or DMSO (**C**) for 72 h followed by fixing and staining with 0.5% crystal violet. The stain was solubilized, and absorbance was read at 570 nm. Data are the mean ± SEM of 3–4 separate experiments expressed as % of control. * *p* < 0.05, *** *p* < 0.001, ns, no significance compared to control. #### *p* < 0.0001 compared to DMSO vehicle control.

H1299 cells were treated with 2.5, 5, 10, 25, 50, 100, 150, or 200 µg/mL of RE followed by assessment of cell proliferation (Figure 1A). A significant (88.4 ± 4.8% of control, non-treated cells, *p* < 0.05) inhibition of proliferation was seen at 2.5 µg/mL RE while concentrations of 25–200 µg/mL resulted in similar (maximum) inhibition of proliferation with a value of 23.3 ± 1.3% of control, *p* < 0.001, seen with 25 µg/mL RE. The calculated concentration of RE to result in half maximal inhibition (IC_50_) was 19 µg/mL.

Interestingly, the inhibition of H1299 cell proliferation with 25 µg/mL RE was similar to the inhibition seen with 100 µM resveratrol (22.3 ± 2.5% of control, *p* < 0.001; Figure 1B), a polyphenol studied extensively and shown to have antiproliferative effects in many cancer cells [33]. Since RE was dissolved in DMSO, we also examined the effect of DMSO (vehicle control) on cell proliferation. We exposed cells to 0.1% DMSO to match the DMSO levels in cells treated with 200 µg/mL RE and found no effect on cell proliferation (Figure 1C) indicating that the inhibitory effects seen with RE treatment were not due to DMSO.

**Figure 2:**
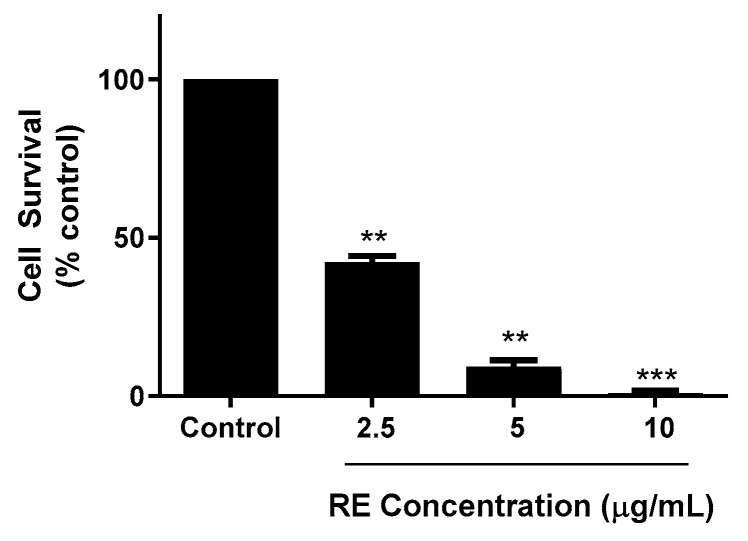
Effect of RE on clonogenic survival of H1299 human lung cancer cells. Cells were seeded in triplicate at a low density and incubated without (control) or with the indicated concentrations of RE for 7 days followed by fixing and staining with 0.05% methylene blue. Colonies of more than 50 cells were counted. The data are expressed as percent of control and are the mean ± SEM of 3–4 separate experiments. ** *p* < 0.01, *** *p* < 0.001, compared to control.

The ability of cancer cells to survive and form colonies was assessed using a clonogenic survival assay [34]. Cells were exposed to different concentrations of RE (2.5, 5, and 10 µg/mL) for 7 days. At the end of the treatment, the cells were fixed and stained, and colonies with greater than 50 cells were counted and compared to control. Colony formation is an indication of the cells’ potential to survive and establish tumour growth in vivo. Significant inhibition of cell survival was seen with 2.5 µg/mL RE (42.3 ± 1.9% of control and near complete inhibition was observed with 10 µg/mL RE (0.8 ± 0.6% of control, *p* < 0.001; Figure 2).

**Figure 3:**
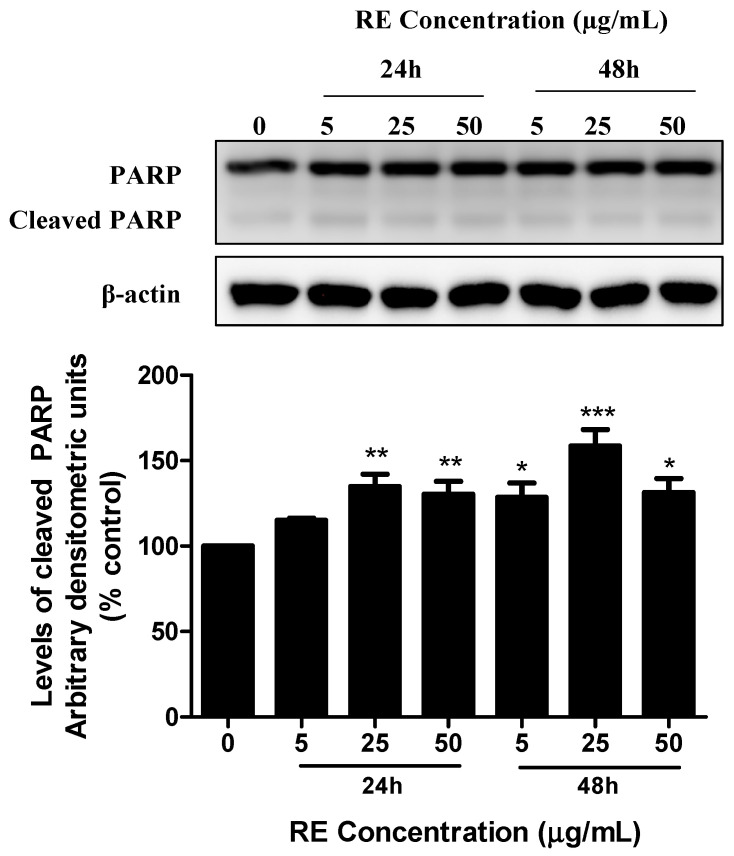
Effect of RE on PARP. Whole cell lysates were prepared from H1299 cells treated without (control) or with the indicated concentrations of RE for 24 h or 48 h. Cell lysates (20 µg) were resolved by SDS-PAGE and immunoblotted with specific antibodies against PARP or β-actin. Upper panel: A representative immunoblot is shown. Lower panel: The densitometry of the bands, expressed in arbitrary units, was measured using ImageJ software. The data are expressed as percent of control and are the mean ± SEM of 3–5 separate experiments. * *p* < 0.05, ** *p* < 0.01, *** *p* < 0.001, compared to control. Original western blot figure can be found in Figure S1.

The cleavage of poly (ADP-ribose) polymerase (PARP) is a known indicator of cell apoptosis. Treatment with RE dose- and time-dependently increased cleaved PARP levels (Figure 3). Following 24 h treatment with 25 µg/mL or 50 µg/mL of RE, the levels of cleaved PARP increased to 134.9 ± 7.2% (*p* < 0.01) and 130.4 ± 7.5% (*p* < 0.01) of control, respectively. Similarly, exposure of H1299 cancer cells to 5 µg/mL, 25 µg/mL, or 50 µg/mL RE for 48 h resulted in a significant increase in cleaved PARP to 128.6 ± 8.3% (*p* < 0.05) 158.6 ± 9.7% (*p* < 0.001) and 131.5 ± 8.1% (*p* < 0.05) of control, respectively (Figure 3).

### 3.2. RE Inhibits Migration of H1299 Lung Cancer Cells

**Figure 4:**
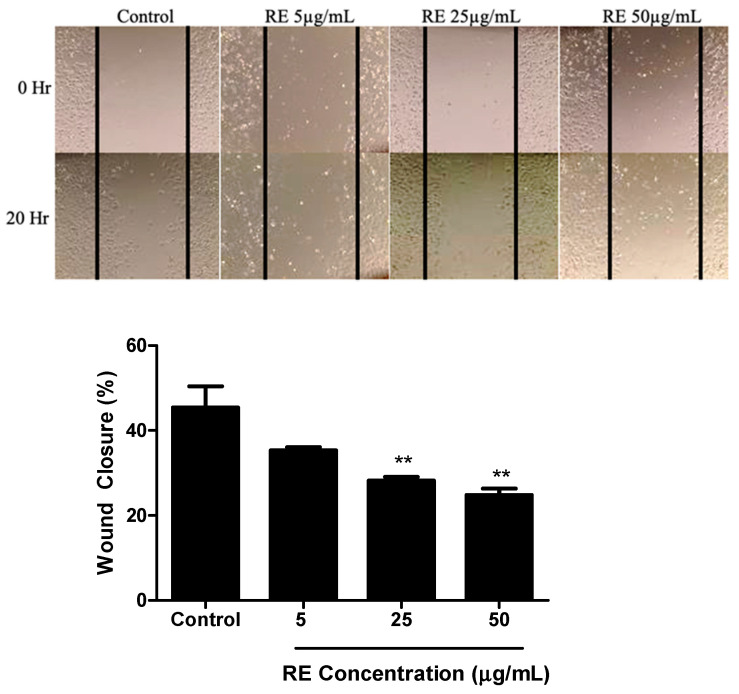
Effect of RE on H1299 human lung cancer cell migration. Confluent H1299 cells were exposed to 1 μg/mL mitomycin-C (MMC) for one hour, followed by a wound/ scratch injury (black lines) and treatment without (control) or with 5 μg/mL, 25 μg/mL, 50 μg/mL RE. The data shown are the mean ± SEM of 3 separate experiments. ** *p* < 0.01, compared to control.

A wound healing assay was used to assess the effects of RE on H1299 NSCLC cell migration. The cells were grown to around 90% confluency then treated with mitomycin-C (MMC) to halt proliferation. This was followed by a scratch/wound of the cell layer down the centre of the well and treatment with 5, 25, or 50 µg/mL RE for 20 h. Control cells migrated and resulted in 45.40 ± 5.0% would closure (Figure 4). At a dose of 5 µg/mL RE exhibited no significant inhibition of migration compared to control. However, RE concentrations of 25 and 50 µg/mL were able to significantly inhibit migration with an observed wound closure of 28.20 ± 0.9% (*p* < 0.01) and 24.82 ± 1.5% (*p* < 0.01), respectively (Figure 4).

### 3.3. RE Increases ERK Phosphorylation in H1299 Lung Cancer Cells

**Figure 5:**
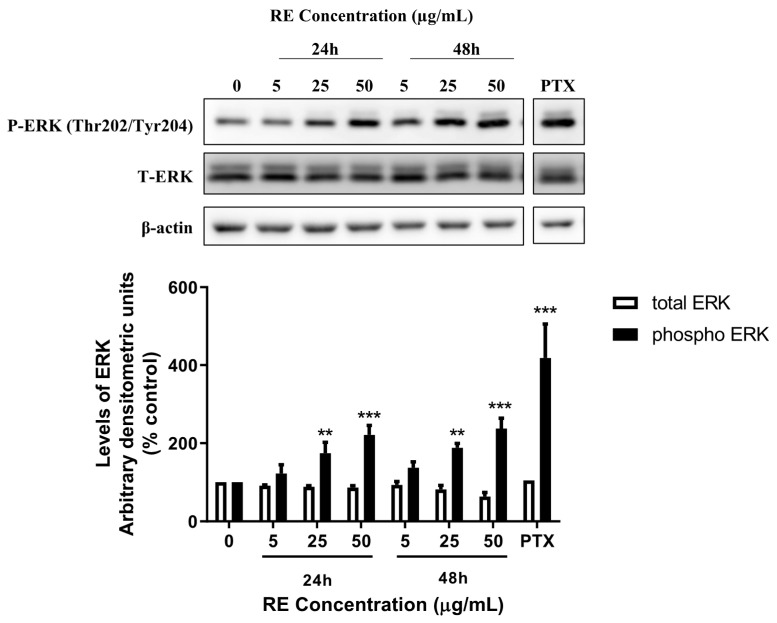
Effect of RE on ERK. Whole cell lysates were prepared from H1299 cells treated without (control) or with the indicated concentrations of RE for 24 h or 48 h, or 50 nM PTX for 48 h. Cell lysates (20 µg) were resolved by SDS-PAGE and immunoblotted with specific antibodies against total or phosphorylated ERK or β-actin. Upper panel: A representative immunoblot is shown. Lower panel: The densitometry of the bands, expressed in arbitrary units, was measured using ImageJ software. The data are expressed as percent of control and are the mean ± SEM of 4–5 separate experiments. ** *p* < 0.01 *** *p* < 0.001, compared to control. Original western blot figure can be found in Figure S2.

Phosphorylation of ERK at the threonine-202 and tyrosine-204 (Thr202/Tyr204) residues is an indication of ERK activation and we used an antibody that recognizes phosphorylation of these residues in an attempt to investigate potential signalling mechanisms involved in the anticancer effects of RE. H1299 cells were treated without (control) or with 5, 25, or 50 µg/mL RE for 24 or 48 h. Phosphorylation of ERK was enhanced significantly following treatment with 25 or 50 µg/mL RE for 24 h (174.3 ± 28.1%, *p* < 0.01; 221.1 ± 24.0% of control, *p* < 0.001, respectively) or 48 h (188.5 ± 10.5%, *p* < 0.001; 237.4 ± 26.2% of control, *p* < 0.001, respectively). No effect on total ERK protein levels was seen by any of the treatments (Figure 5). Paclitaxel (PTX) is a chemotherapy agent commonly used in the treatment of lung cancer and has been shown to activate ERK in other cancer cell lines [35]. PTX was used in the present study as a positive control for ERK activation. Cells treated with 50 nM PTX showed a robust phosphorylation/activation of ERK (418.2 ± 87.7% of control, *p* < 0.001). There was no significant difference in total or phosphorylated levels of ERK between the 24 and 48 h control groups (Figure S3). The original western blot figures can be seen in supplementary material.

### 3.4. RE Increases AMPK Phosphorylation in H1299 Lung Cancer Cells

**Figure 6:**
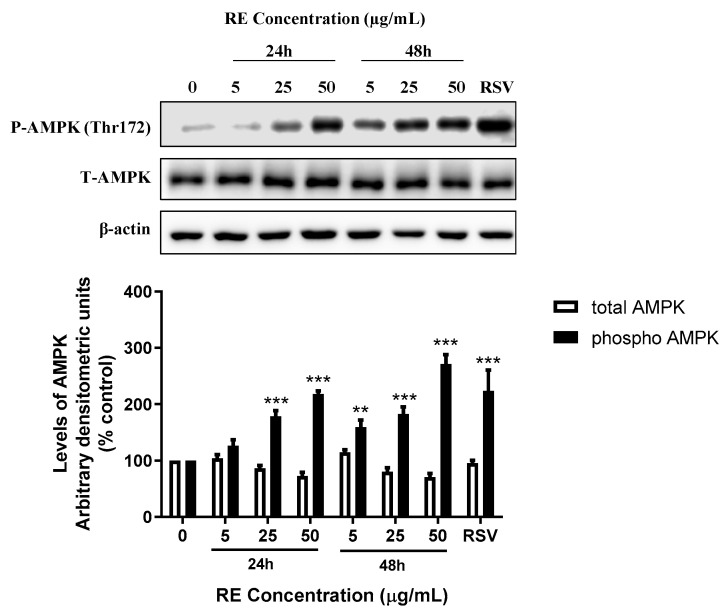
Effect of RE on AMPK. Whole cell lysates were prepared from H1299 cells treated without (control) or with 5, 25, or 50 µg/mL RE for 24 or 48 h, or 10 µM RSV for 48 h. Cell lysates (20 µg) were resolved by SDS-PAGE and immunoblotted with specific antibodies against total or phosphorylated AMPK or β-actin. Upper panel: A representative immunoblot is shown. Lower panel: The densitometry of the bands, expressed in arbitrary units, was measured using ImageJ software. The data are expressed as percent of control and are the mean ± SEM of 3–6 separate experiments. ** *p* < 0.01, *** *p* < 0.001, compared to control. Original western blot figure can be found in Figure S4.

Phosphorylation of AMPK on the threonine-172 residue of its catalytic alpha subunit is an established indicator of AMPK activation, and therefore we used an antibody that recognizes phosphorylation of this residue. H1299 cells were treated without (control) or with 5, 25, or 50 µg/mL RE for 24 or 48 h. RE treatment caused a dose-dependent increase in AMPK phosphorylation with significance seen with 25 and 50 µg/mL RE treatment after 24 h (178.6 ± 10.1%, *p* < 0.001; 218.3 ± 5.2% of control, *p* < 0.001, respectively). Following 48 h of treatment with 5, 25, and 50 µg/mL RE, phosphorylated AMPK levels increased significantly (159.0 ± 13.2%, *p* < 0.01; 182.5 ± 12.6%, *p* < 0.001; 271.1 ± 17.0% of control, *p* < 0.001, respectively). Total AMPK levels were not significantly altered by any treatment (Figure 6).

**Figure 7:**
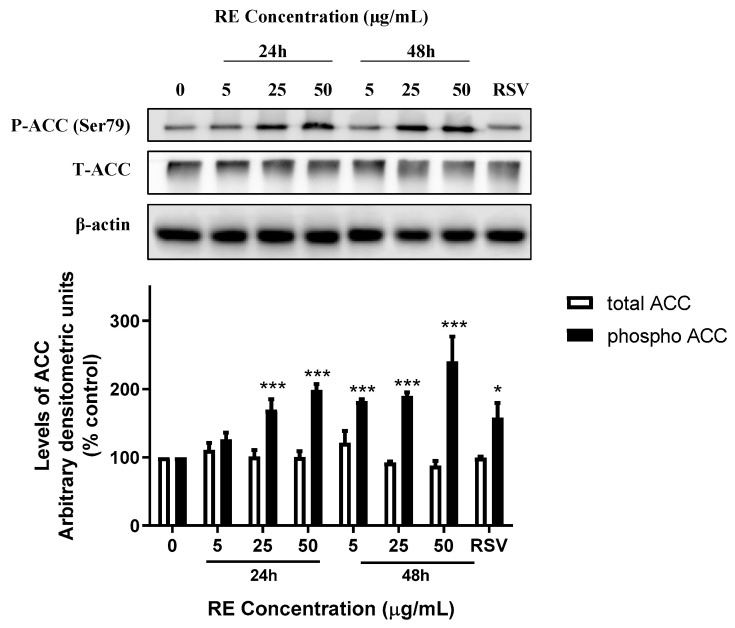
Effect of RE on ACC. Whole cell lysates were prepared from H1299 cells treated without (control) or with 5, 25, or 50 µg/mL RE for 24 or 48 h, or 10 µM RSV for 48 h. Cell lysates (20 µg) were resolved by SDS-PAGE and immunoblotted with specific antibodies against total or phosphorylated ACC or β-actin. Upper panel: A representative immunoblot is shown. Lower panel: The densitometry of the bands, expressed in arbitrary units, was measured using ImageJ software. The data are expressed as percent of control and are the mean ± SEM of 3–6 separate experiments. * *p* < 0.05, *** *p* < 0.001, compared to control cells. Original western blot figure can be found in Figure S5.

Phosphorylation of ACC, a downstream target of AMPK, was also assessed. H1299 cells were treated without (0, control) or with 5, 25, or 50 µg/mL RE for 24 or 48 h. RE treatment caused a significant increase in the levels of phosphorylated ACC after 24 h (25 µg/mL: 169.9 ± 15.1%, *p* < 0.001; 50 µg/mL: 198.7 ± 8.8% of control, *p* < 0.001) and 48 h (5 µg/mL: 182.1 ± 2.9%, *p* < 0.001; 25 µg/mL: 189.8 ± 5.4%, *p* < 0.001; 50 µg/mL: 240.3 ± 36.5% of control, *p* < 0.001), (Figure 7). The total ACC protein levels were not significantly affected by any treatment. The polyphenol resveratrol is known to increase phosphorylation of AMPK and ACC [36,37]. Treatment of cells with 10 µM resveratrol, used as a positive control, led to significant increase in phosphorylation of both AMPK (223.9 ± 36.7% of control, *p* < 0.001; Figure 6) and ACC (158.2 ± 37.5% of control, *p* < 0.05; Figure 7).There was no significant difference in total or phosphorylated levels of AMPK and ACC between the 24 and 48 h control groups (Figure S3). 

### 3.5. RE phosphorylates Raptor in H1299 NSCLC Cells

**Figure 8:**
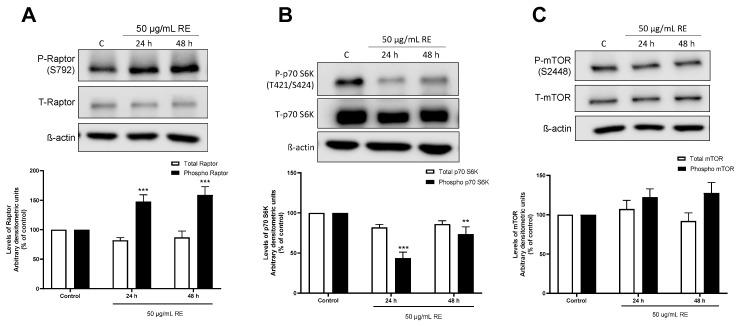
Effects of RE on mTORC1. Whole cell lysates were prepared from H1299 cells treated without (control) or with 50 µg/mL RE for 24 or 48 h. Cell lysates (20 µg) were resolved by SDS-PAGE and immunoblotted with specific antibodies against total or phosphorylated Raptor (**A**), p70 S6K (**B**), mTOR, (**C**) or β-actin. The densitometry of the bands, expressed in arbitrary units, was measured using ImageJ software. The data are expressed as percent of control and are the mean ± SEM of 7 separate experiments. ** *p* < 0.01, *** *p* < 0.001, compared to control cells. Original western blot figure can be found in Figure S6.

AMPK activation leads to direct serine-722/792 phosphorylation of raptor [25] leading to inhibition of mTOR activity. We wished to examine downstream targets of AMPK affecting mTOR activity and measured both phosphorylated (S792) and total raptor. Treatment with 50 μg/mL RE significantly increased phosphorylated levels of raptor (24 h: 147.6 ± 11.5%, *p* < 0.001; 48 h: 159.1 ± 13.8% of control, *p* < 0.001) with no significant change in total raptor levels (Figure 8A).

p70 S6K phosphorylation at thereonine-389 is downstream effector of mTOR1 activation and a readout of p70 S6K activity [23]. RE treatment decreased phosphorylated levels of p70 S6K (24 h: 43.7 ± 7.4%, *p* < 0.001; 48 h: 73.6 ± 9.0% of control, *p* < 0.01) with no change in total p70 S6K levels (Figure 8B). We also examined the effect of RE treatment on phosphorylation of mTOR at serine-2448. Treatment with 50 μg/mL RE for 24 or 48 h had no significant effect on the levels of phosphorylated or total mTOR (Figure 8C).

**Figure 9:**
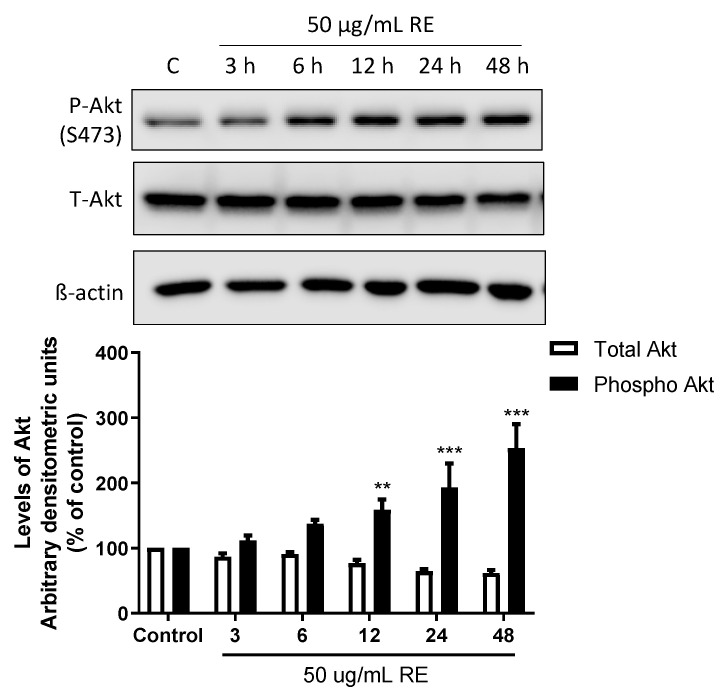
Effects of RE on Akt. Whole cell lysates were prepared from H1299 cells treated without (control) or with 50 µg/mL RE for 3–48 h. Cell lysates (20 µg) were resolved by SDS-PAGE and immunoblotted with specific antibodies against total or phosphorylated Akt with β-actin used as a loading control. The densitometry of the bands, expressed in arbitrary units, was measured using ImageJ software. The data are expressed as percent of control and are the mean ± SEM of 4 separate experiments. ** *p* < 0.01, *** *p* < 0.001, compared to control cells. Original western blot figure can be found in Figure S7.

mTOR is downstream of AMPK and Akt signaling. Activated AMPK inhibits mTORC1 while activated Akt phosphorylates/activates mTOR by inhibiting TSC2 [21,22]. Furthermore, a number of studies have shown that activation of AMPK [38] and inhibition of mTORC1 [39,40] leads to feedback activation of Akt. If such feedback activation of Akt happens with RE treatment, this would explain the lack of an effect on phosphorylated levels of mTOR since Akt and AMPK have opposing effects on mTOR activation. For this reason, we next examined Akt and found that exposure of the cells to 50 µg/mL RE resulted in Akt activation in a time-dependent manner with significant increase in phosphorylation observed after 12 h (158.7 ± 16.0%), 24 h (192.7 ± 37.0%, *p* < 0.001), and 48 h (253.3 ± 36.9%, of control *p* < 0.001; Figure 9).

## 4. Discussion

The H1299 lung cancer cells lack the tumor suppressor p53 and express a mutated constitutively active form of Ras [41]. Our data showed that treatment of H1299 cells with RE resulted in inhibition of proliferation, survival, and migration indicating that RE has anticancer properties in cells expressing activated Ras and lacking functional p53. Previously, RE has been shown to inhibit proliferation in various cancer cell lines [6] and in a previous study by our group we found inhibition of proliferation of NSCLC A549 cells by RE with an IC_50_ of 15.9 µg/mL [29]. RE has also been shown to reduce cell viability in NCI-H82 small cell lung cancer cells with an IC_50_ of 24.08 µg/mL [42]. In the present study, RE inhibited H1299 proliferation with an IC_50_ of 19 µg/mL. Collectively, these data point to antiproliferative effects of RE with an IC_50_ in the range of 15–25 µg/mL. Our data align with the results of similar studies, which examined the antiproliferative effects of RE using similar concentrations in prostate [32] and other cancer cells [6]. Interestingly, the effects of RE at its maximum inhibitory dose of 25 µg/mL were comparable to the extensively studied polyphenol resveratrol which also has significant anticancer effects in vitro [33].

Another characteristic of cancer cells is their enhanced ability to survive and form tumours despite the harsh environments generated by radiation and chemotherapy [8]. The current study showed that clonogenic cell survival was dose-dependently inhibited by RE. Significant inhibition of survival was seen with 2.5 µg/mL and near complete inhibition was seen when treated with 10 µg/mL RE. The present data are in agreement with the inhibition of survival of A549 lung cancer cells [29], and the inhibition of viability—an indicator of survivability—of colon, breast, leukemia, prostate, liver, and small cell lung cancer cells [42] seen with RE treatment at concentrations similar to those used in the current study.

In addition to inhibition of proliferation and survival, the present study found RE to induce apoptosis in H1299 cells as indicated by the increased levels of cleaved PARP. These data are in agreement with other studies that reported RE-induced apoptosis in breast [34], prostate [32], and lung [29] cancer cells. Future studies should aim to identify which apoptotic signalling molecules are modulated by RE upstream of PARP cleavage in order to better understand how RE induces apoptosis in cancer cells.

Enhanced cell migration is a common characteristic of cancer cells which contributes to metastasis [8]. In the current study, treatment with RE caused significant inhibition of H1299 cell migration. Although the inhibitory effect of RE on cancer cell migration has not been studied extensively, in agreement with our data, significant inhibition of migration was seen with RE (30–50 µg/mL) treatment of prostate [32], and breast [34] cancer cells. The ability of RE to inhibit migration and reduce clonogenic survival suggests a potential to inhibit metastasis of NSCLC.

Phosphorylation of ERK is an established indicator of its activation and an activated Ras-Raf-MEK-ERK signalling cascade is generally associated with increased proliferation, survival, and metastasis, but also with promoting antiapoptotic proteins such as Bcl-2, and inhibiting proapoptotic proteins such as Bad [43]. Furthermore, activation of ERK can contribute to enhanced invasion and migration of cancer cells by activating the transcription factor AP-1, which in turn increases transcription of MMPs, which are responsible for disrupting the extracellular matrix allowing for cancer cells to invade surrounding tissues [44]. Small molecule inhibitors of ERK signalling have shown some promise as anticancer agents [9]. In the present study we found inhibition of cell proliferation, survival, and migration and induction of apoptosis with RE treatment that corelate with increased phosphorylation/activation of ERK. Similar to our findings RE induced apoptosis in colon cancer cells through a nuclear factor erythroid 2-related factor 2 (Nrf2)/sestrin-2 pathway, which required phosphorylation/activation of ERK [45]. Increased ERK phosphorylation and apoptosis was observed in p53-null HeLa and HL-60 cancer cell lines following treatment with the anti-cancer drug cisplatin [46]. The activation of ERK, as seen by increased pERK levels, in the present study, could be a mechanism leading to apoptosis similar to other studies [45,46].

In the present study, we found a robust phosphorylation of AMPK by RE treatment. In addition, the phosphorylation levels of ACC, the downstream target of AMPK, were significantly increased confirming activation of AMPK. It is unclear how RE causes phosphorylation/activation of AMPK. RE components could be directly phosphorylating AMPK, activating signaling molecules upstream of AMPK, or inducing ATP deprivation. H1299 cells expresses wild type LKB1 and the RE-induced phosphorylation of AMPK may be LKB1-dependent. Alternatively, RE may lead to AMPK phosphorylation by increasing ROS generation and reducing ATP levels as seen in A549 NSCLC cells by sarcosine treatment [47].

Similar to our data, other studies have found enhanced apoptosis of cancer cells that correlated with increased phosphorylation/activation of AMPK [48,49,50]; however, the exact mechanism linking AMPK activation with apoptosis is not entirely understood. Experiments using multidrug treatment of colon cancer cells indicated that AMPK activation promoted apoptosis by phosphorylating Beclin-1 at Ser93/96, which increases Beclin-1 cleavage by caspase-8 [50]. Cleavage of Beclin-1 promotes the release of proapoptotic factors from the mitochondria which in turn activate caspases that cleave PARP, thereby triggering apoptosis [50]. Other studies have found anticancer effects of various naturally-derived compounds that were associated with AMPK activation in NSCLC [51,52,53]. Treatment of lung cancer cells (HOP62 and H1975) with the polyphenol ellagic acid resulted in AMPK activation and reduced cell proliferation [51]. Oral administration of ellagic acid reduced the growth of Lewis Lung Carcinoma (LLC) xenografts in mice and was associated with increased levels of phosphorylated AMPK and ACC in tumour tissues [51]. Treatment of A549 NSCLC cells with a synthetic derivative of the polyphenol curcumin inhibited proliferation and migration, and these effects were associated with increased phosphorylation/activation of AMPK [52]. The natural compound phillygenin from the medicinal herb *Forsythia suspensa,* activated AMPK, inhibited growth, and promoted apoptosis of A549 and SPC-A1 NSCLC cells in vitro and administration of phillygenin to A549 xenografted nude mice resulted in reduced tumour volume [53].

Two isoforms of ACC exist in human tissues, and AMPK inhibits this molecule by phosphorylating serine 79, 1200, and 1215 residues on ACC1 and serine 222 on ACC2 [54,55]. ACC is the rate limiting enzyme for malonyl-CoA synthesis, which leads to inhibition of fatty acid oxidation. Thus, following activation of AMPK and subsequent inhibition of ACC, malonyl-CoA synthesis is decreased leading to enhanced fatty acid oxidation and ATP production, restoring cellular energy levels [56]. In many cancer cells, the activation of ACC is upregulated and therefore these cells have elevated rates of fatty acid synthesis [57]. In NSCLC cells, ACC is highly expressed which suggests NSCLC cells highly depend on fatty acid synthesis by ACC to promote growth/survival [57]. The increase in AMPK phosphorylation/activation by RE treatment could explain the subsequent phosphorylation and inhibition of ACC seen in H1299 cells in the present study. Alternatively, RE may affect ACC directly. Our data suggest that ACC phosphorylation by RE contributes to its anticancer effects.

Another downstream target of AMPK is mTOR. Increased AMPK activation has been shown to inhibit proliferation by inhibiting mTOR activation [58]. Previous studies from our lab have shown that RE inhibits the phosphorylation/activation of mTOR in PC-3 prostate cancer cells, MDA-MB-231 breast cancer cells, and A549 NSCLC cells [29,32,34]. Examination of mTOR phosphorylation did not reveal any changes with RE treatment in the current study. As mentioned in the introduction mTOR exists in two different complexes mTORC1 and mTORC2. It is likely that mTOR present in mTORC1 is inhibited by RE treatment but we could not detect it because we utilized an antibody that recognizes serine-2448 phosphorylation of mTOR present in both mTORC1 and mTORC2. Another possibility is that the lack of changes in mTOR phosphorylation (Figure 8) is due to antagonistic effect of AMPK and Akt on mTOR. TSC2, an upstream signaling molecule that inhibits mTOR, is activated by AMPK and inhibited by Akt [21,22,25]. Therefore, activation of AMPK and Akt, as is the case with RE treatment, has opposing effects on mTOR resulting in no net change in mTOR phosphorylation status.

In the current study we found increased phosphorylation of raptor (confirming activation of AMPK) and inhibition of phosphorylation of p70 S6K indicating inhibition of mTORC1. Activated AMPK directly phosphorylates raptor on serine-722/792 leading to inhibition of mTORC1 activity and its downstream target p70 S6K [24,25]. Collectively, our data indicate that the inhibition of p70 S6K phosphorylation/activation is mediated by AMPK activation and mTORC1 inhibition is mediated by increased raptor phosphorylation.

Our data indicated a time-dependent increase in phosphorylated/activated levels of Akt following RE treatment. Activated p70 S6K inhibits Akt through a feedback mechanism involving IRS/PI3K signalling [59], and therefore the increased activation of Akt may be explained by the inhibition of p70 S6K seen with RE treatment. In addition, evidence indicate that increased AMPK activation leads to activation of Akt. AMPK activation using AICAR [38] and mTORC1 inhibition using rapamycin [39,60] or raptor-siRNA [40] were found to cause compensatory activation of Akt despite inhibition of cell survival. It is possible that a similar process is occurring in H1299 cells following RE treatment. Other studies have shown that activation of Akt enhances ROS-mediated apoptosis [61] and the proapoptotic effects of rosemary have been attributed—at least in part—to induction of ROS in some cell lines [62,63,64,65], so it is possible that activation of Akt, seen with RE treatment in our study, is a mediator of apoptosis.

Future studies should employ small interference RNA (siRNA) techniques or specific inhibitors to elucidate the roles of ERK, AMPK, mTOR, and Akt in RE-induced anticancer effects.

We acknowledge that any plant extract, including the RE prepared in our lab, contains many chemicals/compounds, and it is extremely difficult to identify the exact compounds responsible for the observed biological effects of the extract. A search of the literature revealed studies indicating the predominant presence of the polyphenols rosmarinic acid (RA) and carnosic acid (CA) in RE [7]. In addition we found studies pointing to anticancer properties of RA and CA when used alone in in vitro models of NSCLC [66,67]. The potent anticancer effects seen with RE treatment could be attributed to CA, RA or any of the many chemicals present in RE each acting alone or in combination/synergistically.

## 5. Conclusions

**Figure 10:**
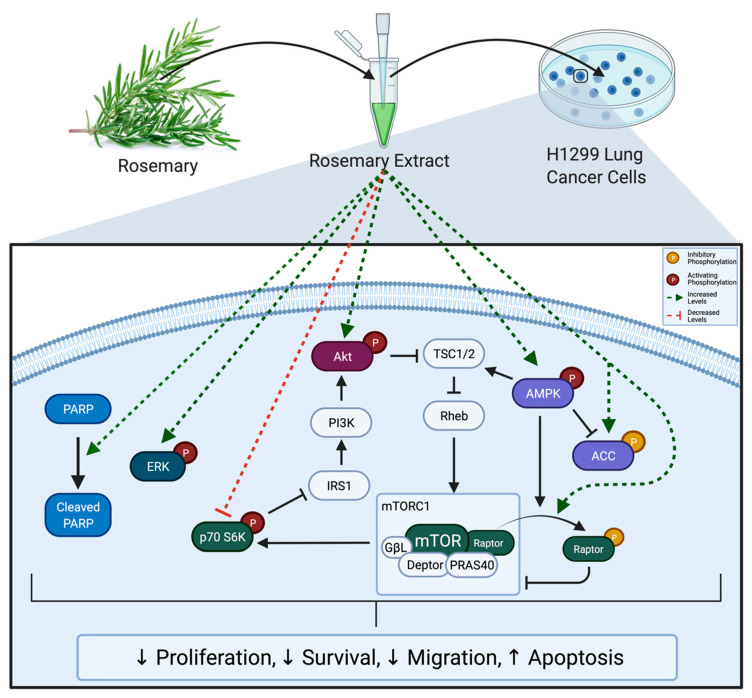
Summary of the effects of RE on H1299 cells. Rosemary extract decreased proliferation, survival, and migration; and enhanced apoptosis of H1299 lung cancer cells. These effects were associated with increased PARP cleavage; increased ERK, Akt, AMPK, ACC, and Raptor phosphorylation; and decreased p70 S6K phosphorylation.

The present study is the first to show significant inhibitory effects of RE treatment in H1299 human lung cancer cells. Treatment with RE significantly reduced proliferation, survival, and migration of H1299 cells. Additionally, treatment with RE induced apoptosis as indicated by increased levels of cleaved PARP. RE increased phosphorylation/activation of ERK and AMPK. Downstream of AMPK, ACC phosphorylation/inactivation increased with RE treatment as did phosphorylation/inactivation of the mTORC1 protein raptor. mTORC1 inhibition was confirmed by decreased levels of phosphorylated p70 S6K. Overall, these data suggest that RE possesses significant anticancer properties and that the mechanism of action of RE may involve activation of ERK and AMPK signalling, and inhibition of mTORC1 (Figure 10). Future studies should aim to identify if ERK and AMPK activation are required for the observed proapoptotic and antiproliferative effects of RE, as well as seeking to identify how RE causes activation of these molecules, and which compounds present in RE confer its anticancer properties.

## Data Availability

The data presented in this study are available within the article and supplementary materials.

## Supplementary Materials

The following supporting information can be downloaded at: https://www.mdpi.com/article/10.3390/life12010052/s1, Figure S1: Original, unedited PARP and β-actin representative blots corresponding to Figure 3; Figure S2: Original, unedited ERK and β-actin representative blots corresponding to Figure 5; Figure S3: Representative blots with control untreated cells at 24 and 48 h; Figure S4: Original, unedited AMPK and β-actin representative blots corresponding to Figure 6; Figure S5: Original, unedited ACC and β-actin representative blots corresponding to Figure 7; Figure S6: Original, unedited Raptor, p70 S6K, mTOR, and β-actin representative blots corresponding to Figure 8; Figure S7: Original, unedited Akt and β-actin representative blots corresponding to Figure 9.
