# Supplementary material for: Inhibition of Non-Small Cell Lung Cancer Proliferation and Survival by Rosemary Extract Is Associated with Activation of ERK and AMPK
Source: Life (Basel). 2021 Dec 31;12(1):52. doi: 10.3390/life12010052 (PMC8779065; doi:10.3390/life12010052)
Supplement: Supplementary file 1 [file life-12-00052-s001.zip › life-1329507-supplementary.pdf]

life

Supplementary Materials

Inhibition of Non-Small Cell Lung Cancer Proliferation and Survival by Rosemary Extract is Associated with Activation of ERK and AMPK

Eric J. O'Neill, Jessy Moore, Joon Song, and Evangelia Litsa Tsiani

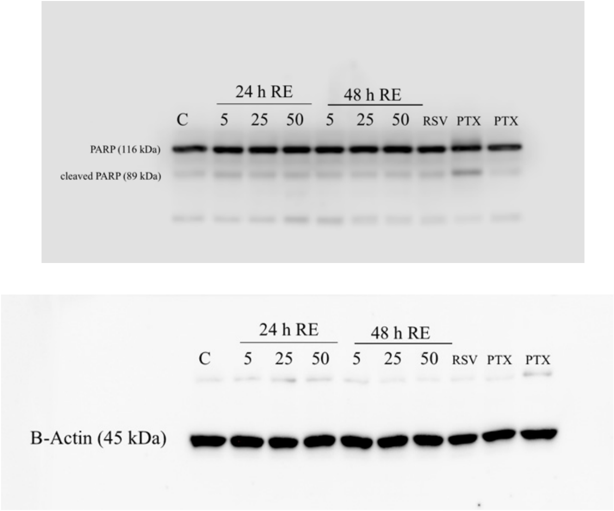

Figure S1. Original, unedited PARP and β-actin representative blots corresponding to Figure 3.

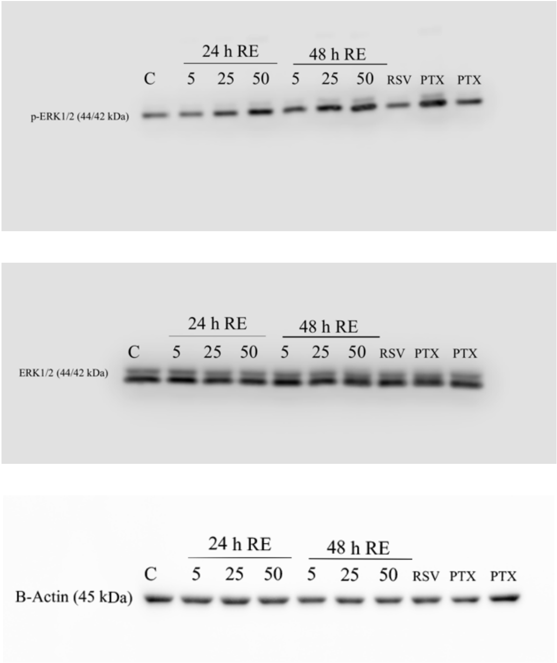

Figure S2. Original, unedited ERK and β-actin representative blots corresponding to Figure 5.

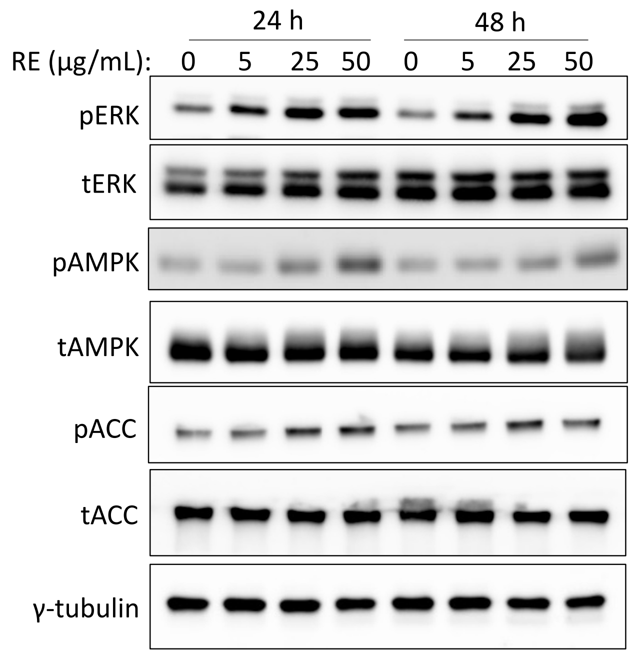

Figure S3. Representative blots with control untreated cells at 24 and 48h. Whole cell lysates were prepared from H1299 cells treated with 0, 5, 25, 50 μg/mL RE for 24 and 48 h. Each timepoint had a separate control group. Cell lysates (20 μg) were resolved by SDS-PAGE and immunoblotted with specific antibodies against total or phosphorylated ERK, AMPK, and ACC with γ-tubulin used as a loading control.

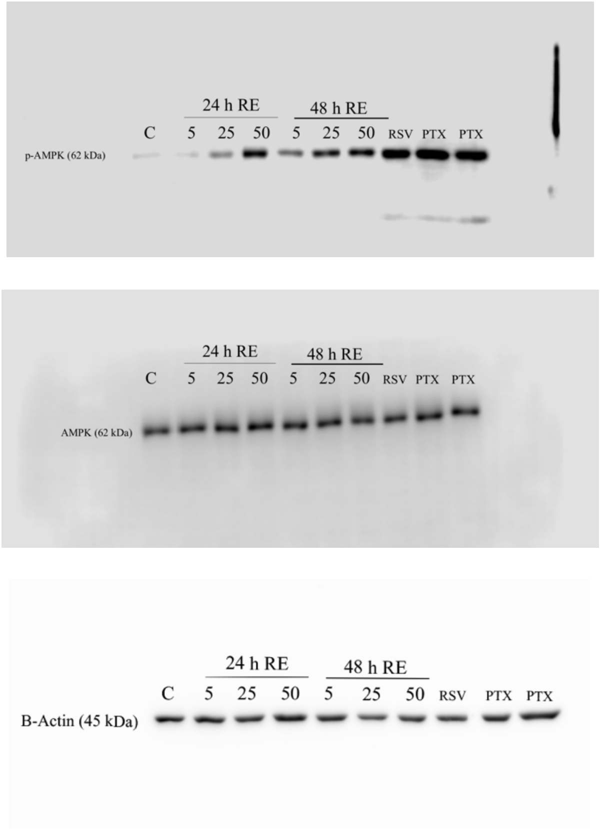

Figure S4. Original, unedited AMPK and β-actin representative blots corresponding to Figure 6.

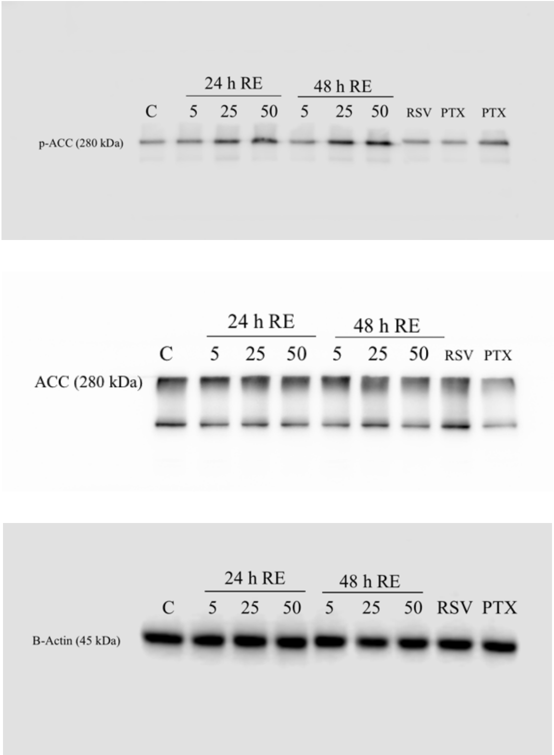

Figure S5. Original, unedited ACC and β-actin representative blots corresponding to Figure 7.

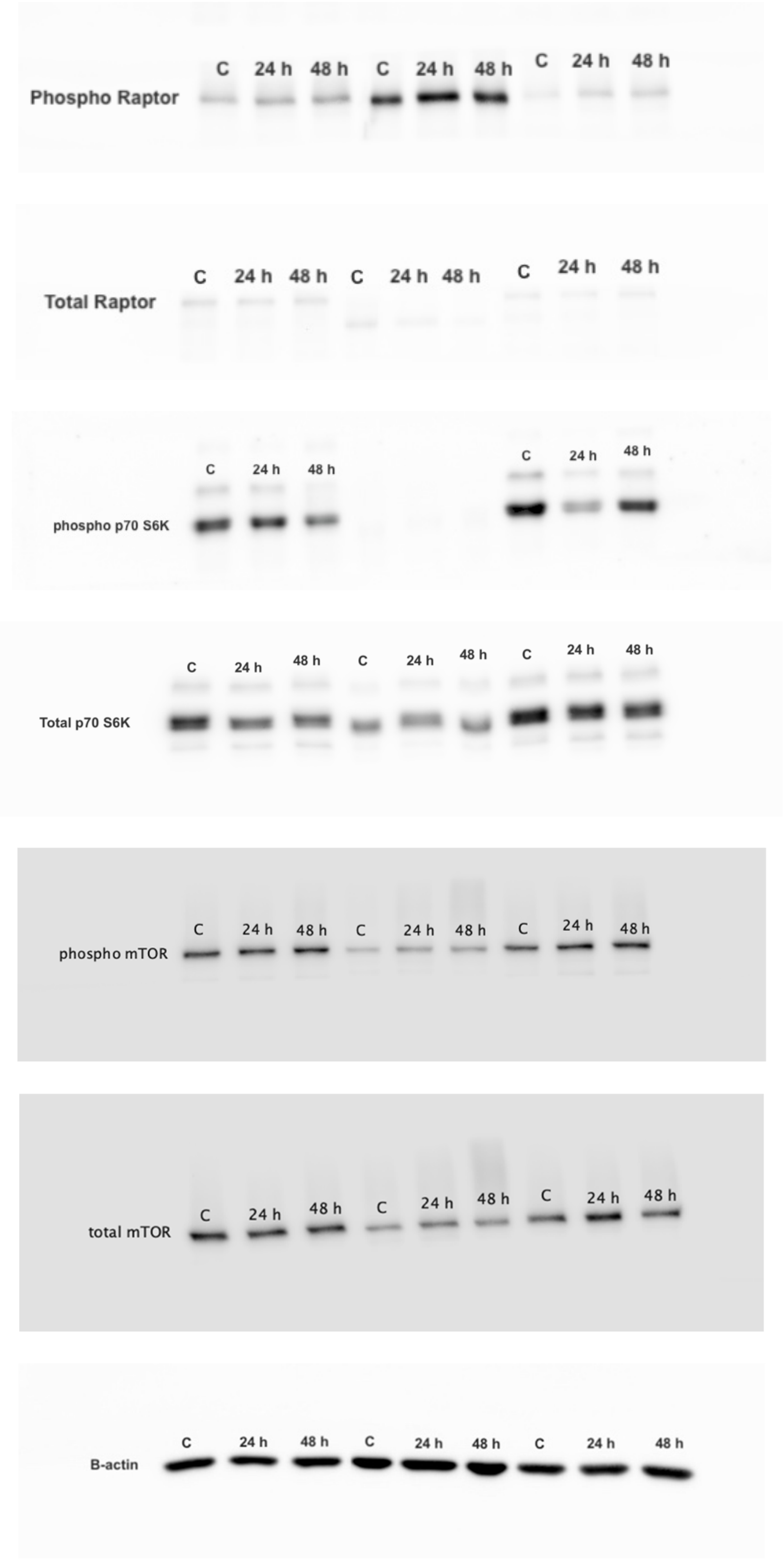

Figure S6. Original, unedited Raptor, p70 S6K, mTOR, and β-actin representative blots corresponding to Figure 8.

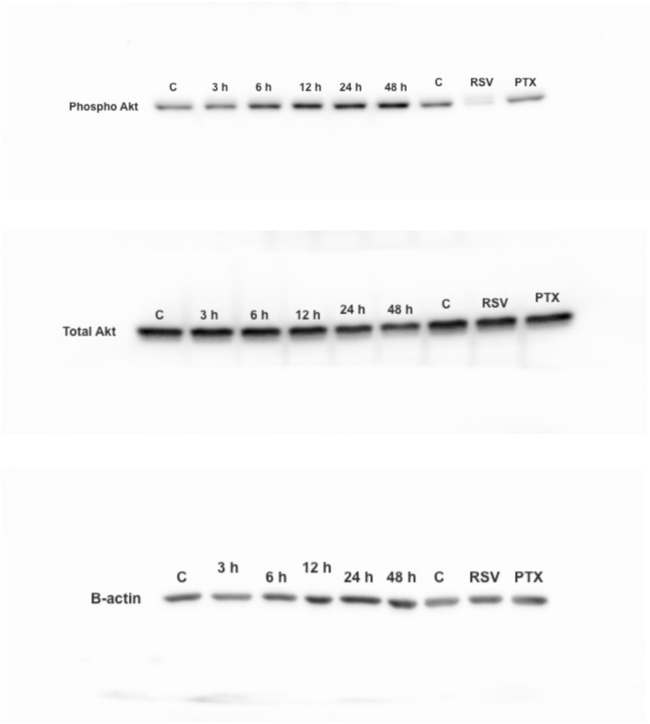

Figure S7. Original, unedited Akt and β-actin representative blots corresponding to Figure 9.
